# A Backwoodsman in Philadelphia

**Published:** 1834

**Authors:** 


### XVI.—A Backwoods'-man in Philadelphia.

Dr. Joseph N. McDowell, for three or four years a lecturer on Anatomy in Cincinnati and Lexington, has sought an ampler theatre in the East, and is about to commence a fall course of anatomical lectures in Philadelphia, to be followed by another in the winter. Dr. McDowell is a native of Kentucky, and was never out of the Backwoods till he went to the great emporium of the medical sciences, for the purpose of teaching, instead of being taught. As far as we know, he is the first native of the West, who has had the rashness to engage in such an enterprize. We hope the students of the Great Valley who may be there, will give him a "fair chance," and they will soon discover, that he has a "fine chance" of fluent speech and fervent feeling, with a copious knowledge of anatomy—acquired chiefly by his own dissections. His success at home, in drawing students around him, and making them feel, that they were instructed, is without a parallel in the United States, considering the disadvantages under which he commenced and prosecuted his labors as a public teacher. His late pupils and numerous western friends, will watch with interest his progress among the great men of the East.

